# Advanced practitioners working with older people in primary care and community settings: a survey of roles and use of technology

**DOI:** 10.12968/ijap.2023.0048

**Published:** 2024-10-02

**Authors:** Samantha Febrey, Julia Frost, Abi J Hall, Naomi Morley, Julie Whitney, Vicky Johnston, Payal Wilson, Cliff Kilgore, Victoria A Goodwin

**Affiliations:** Research Assistant, Faculty of Health and Life Sciences, University of Exeter, England; Associate Professor in Health Services Research, Faculty of Health and Life Sciences, University of Exeter, England; Senior Research Fellow, Faculty of Health and Life Sciences, University of Exeter, England; Research Fellow, Faculty of Health and Life Sciences, University of Exeter, England; Lecturer in Physiotherapy, King’s College London; Consultant Practitioner in Gerontology, Kings College Hospital, England; First Contact Physiotherapist (Frailty), Copeland Primary Care Network, England; Advanced Clinical Practitioner, North Manchester Crisis Response Team, Manchester Local Care Organisation, England; Visiting Fellow, Bournemouth University; Consultant Practitioner Integrated Care/Older People, Dorset Healthcare University NHS Foundation Trust, England; Professor of Ageing and Rehabilitation, Faculty of Health and Life Sciences, University of Exeter, England

**Keywords:** community, frailty, primary care, survey, technology

## Abstract

**Background:**

Advanced practitioner (AP) roles are becoming increasingly common in primary care and community settings for supporting older people and those living with frailty.

**Aims:**

The aim of this study was to explore health and social work AP roles in primary care and community settings in the UK, and understand how they support older people and factors that may impact on APs use of technology in practice.

**Methods:**

A cross-sectional, web-based survey was adopted to explore the views and perspectives of APs.

**Results:**

The survey received 111 responses. There were different views as to whether technology was suitable for older people. Although digital exclusion was a concern, it was perceived that this would change in the future as generations become more digitally literate. Respondents suggested that using technology brought about efficiencies and the ability to respond sooner to symptom changes, with some concerns mentioned regarding the accuracy of technology that may miss signs and symptoms.

**Conclusion:**

This is the first national survey to explore advanced practice roles with older people in non-hospital settings. While APs have wide-ranging skills, few currently use technology in their practice. Findings will inform a future study on digitally enhanced comprehensive geriatric assessments.

**T**he population is ageing, and people are living longer with multiple health problems, frailty and disability (Office for National Statistics, 2018). This has resulted in an increased demand for health and care services for those with frailty, compared to older people without frailty (Han et al, 2019). Coupled with workforce shortfalls, there is now considerable pressure on the UK health system; new approaches to health care delivery are essential for future sustainability. The NHS Long Term Plan (NHS England, 2019) focusses on transforming the workforce to address these challenges to enhance provision and improve patient outcomes. Advanced practice roles are conducted by ‘experienced, registered health and social care practitioners with a high degree of autonomy and complex decision making’ (Health Education England, 2017; British Association of Social Workers (BASW), 2018a). The roles are undertaken by those from a range of professions, including nurses, occupational therapists, physiotherapists, pharmacists, paramedics and social workers. Advanced practitioners (APs) contribute to the workforce transformation agenda, enabling career development opportunities and helping to meet the needs of older people (BASW, 2018b; Evans et al, 2020). The roles have been found to result in high patient satisfaction and service efficiency (Bonsall and Cheater, 2008). A survey of AP healthcare roles in England was undertaken in 2019; it received responses from over 4000 advanced clinical practitioners (ACPs) and 352 primary and secondary care organisations (Fothergill et al, 2022). The survey found that there was considerable heterogeneity in roles, scope of practice and governance, and recommended that a more standardised approach to advanced practice workforce development that would enable individuals to work across all four pillars of practice (clinical practice; leadership and management; education; and research).

Allied health professionals (AHPs), pharmacists and nurses have been highlighted as being vital for the delivery of current and future primary and community care, particularly for older people and those living with frailty (Oliver, 2015; Delgado-Silveira and Bermejo-Vicedo, 2021). The Additional Roles Reimbursement Scheme (ARRS) provides funding for these roles to support primary care networks, which aims to enable proactive, personalised and more integrated care (NHS England, 2019). The NHS Long Term Plan (2019) recognises the potential for APs (NHS, 2019). The authors’ public and professional advisory group highlighted the skills and attributes required to undertake assessments of those with complex needs that meet the definition of the AP role (Health Education England, 2017). While there is a growing body of research on APs, there is a need to learn more about these roles, specifically in relation to the assessment and management of older people living with frailty in primary care and community settings.

The authors recently completed an NIHR-funded programme development grant to develop Digital and Remote Enhancements to comprehensive geriatric Assessment and Management (CGA) in primary care and community settings, known as the DREAM study (NIHR203293). DREAM involved three key work streams:

◼ A series of scoping reviews to identify valid wearable and digital technologies that are feasible for use in community settings and could inform a CGA
◼ Interviews with older people and healthcare professionals
◼ Extensive and meaningful engagement with a diverse advisory group of older people living with frailty, family members and carers, and health and social work professionals who helped shape the research.

A key area emphasised in DREAM’s public and professional advisory group workshops was the workforce needed to deliver enhanced CGA. The group highlighted elements of low satisfaction in terms of staff meeting their, often complex, needs, poor efficiency and a lack of continuity. While there has been some focus on digital inclusion Among the older population (Mouland, 2018), there has been less of a focus on staff. It has been recognised that staff do not always have the knowledge, skills or confidence to use digital and remote technology (NHS Digital, 2022). As a direct result of issues raised by the DREAM study advisory group, the authors successfully applied for funding from the NIHR School for Primary Care Research. The funding was allocated for a survey of APs, with aimed to provide insight into a group of health and care professionals who may be key to delivering CGA in primary care and community settings in the future.

## Aims

The aim of this study was to understand current roles and practice of APs working with older people living with frailty in primary care and community settings in the UK. The objectives were to:

1. Identify the characteristics of APs working with older people living with frailty
2. Better understand their scope and roles
3. Explore the current and potential use and challenges of digital and remote approaches to assessment, monitoring and management, and understand their training and support needs
4. Understand what training or support they would need to best use technology with their patients.

## Methods

### Study design

A cross-sectional web-based survey was undertaken using the Jisc Online Surveys (formerly Bristol) online survey tool (Jisc, 2023). Ethical approval was obtained from the University of Exeter Medical School and the Health and Care Professions Research Ethics Committee (Reference 523529). The survey was open from 15 December, 2022, to 6 March, 2023.

### Materials and procedures

An information sheet, including a GDPR statement and inclusion criteria, was provided to the participants at the start of the survey. To complete the survey, individuals confirmed they were a professionally registered health or social work professional, working at an AP level with older people, or people with frailty, in primary care or community settings within the UK. The survey included questions on professional background, qualifications and role, service model, current use of technology within the role/service, opinions of future use of technology, and demographics. Initial questions were developed from records of advisory group discussions, after having reviewed literature on advanced practice roles. These were then refined with the advisory group and piloted, to ensure questions were relevant and clear, and that response options were appropriate (Cobern and Adams, 2020). The survey included both closed questions, requiring a tick box, multiple choice or Likert rating scale as a response, and open questions were included to provide the opportunity for free-text answers. All questions were optional, and answers could be amended during the completion of the survey. Respondents were able to select ‘other’ as an answer to all closed questions, which then provided space for a required free-text response. Where applicable, there was also the option to select ‘rather not say’ or ‘none of the above’. Respondents consented to participate by clicking the final submit button once the survey was completed. Survey responses were anonymous, unless contact details were provided for the purpose of study updates, other research opportunities and entry the prize draw competition. These details were stored securely and separately to the survey responses. Of those that gave their contact details, one was randomly selected to win a £50 voucher of their choice. All data were anonymised for analysis.

A combination of opportunistic and snowballing sampling was used for survey distributions, utilising networks and contacts of the research team and advisory group, regional and national professional networks, professional bodies, special interest groups and social media. Prominent health and social work professionals were tagged as key influencers on social media, and the survey was promoted regularly through posting and re-posting to improve response rate. Emails were sent to network gatekeepers to share with members/teams, along with a recruitment poster containing a QR code to access the survey.

### Public and stakeholder involvement and engagement

The research question was identified by the DREAM study advisory group, which comprised of 10 older people living with frailty and their family members, and a further eight multidisciplinary health and social work professionals. They highlighted the need to clarify the roles and attributes of health and care professionals that may be essential to effectively engage with older people and contribute to CGA. The groups helped develop the survey questions and distribution strategy, assisted with data interpretation and the development of the dissemination strategy. Three members of the multidisciplinary advisory group, who met the study inclusion criteria, piloted a draft online version of the survey. Minor recommendations were proposed, and changes made prior to distribution.

### Data analysis

Prior to analysis, all data were checked for any data irregularities. All responses were valid, no exclusions were made and there were no obvious duplicate entries. Descriptive statistics (mean, standard deviation, frequencies and percentages) were calculated for closed questions using either Microsoft Excel or the analysis function provided by the Jisc platform (Jisc, 2023).

When free-text responses were provided to questions with ‘other’ as a response, the authors grouped answers to form a new category or added to an existing category (where possible). Open question responses were analysed across the dataset (rather than focussing on open responses to individual questions) (Braun et al, 2021) using thematic analysis (Braun and Clarke, 2006). Data were downloaded into Microsoft Excel, where each row represented an individual respondent, and each column represented a specific research question. Data were initially coded to establish the spread and depth of responses, and then categorised using the Non-adoption, Abandonment, Scale up, Spread and Sustainability (NASSS) framework (Greenhalgh et al, 2018). They were then divided into six domains: condition; technology; value proposition; adopters; organisation; and external context. This approach enabled an explanation of the complexity of using using technology in practice. The survey methods and results are reported in this article, and have been done so according to the Checklist for Reporting Results of Internet E-surveys (CHERRIES) (Eysenbach, 2004).

## Results

### Advanced practitioner roles and the services they work in

A total of 111 responses were received from participants. Respondents were based in England (90%), Scotland (6%) and Wales (4%). The completion rate for the whole survey was 41% (45 out of the 111 participants completed all the questions in the survey); the completion rate for all the closed questions (making up 76% of the survey) was 97%. Table 1 and Table 2 provide details of participants and their advanced practice roles. The DREAM advisory group highlighted the need for health and care staff to have advanced knowledge and skills to meet their complex needs; most respondents (83%, 91/110) reported a range of advanced clinical skills such as medicines management, requesting and interpreting medical investigations, and giving injections. They also reported a range of non-clinical activities, such as leadership and management (85%, 93/110), teaching and education (89%, 98/100), and research (39%, 43/110). Most people received supervision, in most cases 56% (62/110) from a medically qualified clinical lead. Participants worked across a variety of services, locations and structures (Table 3). Where reported, teams were almost all multidisciplinary (96%, 107/111).

### Using technology in practice

The authors asked particiapants about use of technology for communication, monitoring and assessment, and intervention delivery (Table 4). Remote monitoring systems such as Whzan Digital Health and DocoboTM were also reported as being used for monitoring a range of clinical signs and systems in two services.

The authors used the NASSS Framework (Greenhalgh et al, 2018) to understand how the free-text data related to the six framework domains (condition; technology; value proposition; adopters; organisation; and the external context), to explicitly consider perspectives on current practice and technology use, and identify areas for future development.

### Condition

Some respondents provided broad statements about older people not being able to use technology. Others provided more detail about some of the health complexities and physical and cognitive impairments that this population may experience, and suggested that these could be barriers to technology use. This included conditions like arthritic hands that affects ability to use small devices such as a smart phone, as well as sensory impairments that could affect communication. Some suggested that frailty was a condition that influenced a person’s ability to be able to understand, engage and adopt technology:

‘Not suitable for all patients; for example, the monitor is handheld, so not all patients can make effective contact with the electrodes due to tremor, cognitive issues or post stroke disabilities.’ Nurse, Community trust

However, others felt that technology could enable greater access to support and care, and provide reassurance, in particular when patients might have difficulty attending a clinic-based appointment:

‘I think remote technology would help improve monitoring of frail older people and also help alleviate their anxiety.’ Nurse, GP practice

‘Patients living remotely and/or with frailty/disability post-stroke, or who are unable to easily physically see their GP in the GPs practice can be assessed within their own home.’ Nurse, Community trust

While there was an awareness of the potential use of technology in health and care, it was acknowledged that accessibility was not equal for all. For some patients, costs may be prohibitory to its adoption into practice:

‘The technology is already there, but it’s expensive, such as the Apple Watch, and as technology is rapidly accelerating, there are older people who may be late adopters of technology and less computer literate.’ Physiotherapist, Mixed-acute community trus

Many expressed the view that older people do not currently have the skills, understanding or confidence to use technology:

‘Older people may not be able to use digital technology, or experience a lack of awareness and confidence accessing them.’ Physiotherapist, Acute trust

However multiple respondents felt that digital literacy would become less of a barrier in the future, as the next generation of older people will have had greater exposure to technology throughout their lives:

‘I think that as the generation changes to one of which embraces and has used technology almost daily, that it will continue to be used, and will make a massive different to people. Ease of getting access to tests and investigations without as much interruption to daily life.’ Occupational therapist, Mental health trust

‘At present, I think it is difficult, as this generation haven’t grown up with technology, so are often unable to use it. However, I think future generations will be able to access these services more when that time arises.’ Physiotherapist, GP practice

### Technology

Respondents highlighted the need for technology to be easy to use and appropriate for the user. They also stated that technology could also include features such as voice activation or artificial intelligence- (AI) driven sensors:

‘Some patients will find use of technology daunting, but, if applications are simple and they are given a bespoke ipad, [they] should be able to use [them] effectively.’ Nurse, Acute trust

The two main areas highlighted for future technological use were around clinical observations—such as mobility sensors or basic observations—and remote contact, such as video consultations and assessments:

‘I think [it would be better for patients] if [they] had easy access [to] something as easy as an Alexa-type of function for them to get data, contact the surgery if they have issues with hearing, or contact for spouses who are able to report progression of those who are unwell, [patients could be] support[ed] to remain at home.’ Nurse, GP practice

‘Increased role to support people at home; more use of virtual wards with health monitoring equipment, and remote ward round and consultations.’ Occupational therapist, Primary care network

### Value proposition

Many APs had the view that technology could improve service delivery from both the staff and patient perspective, thus its potential value lay in both efficiency and acceptability. Travel time for staff and patients could be reduced, and unnecessary face-to-face visits could be avoided; such measures could save time and money, and improve healthcare access. Reported views were that it could allow for closer and more frequent monitoring, and improve the communication of more patients and ease isolation:

‘Saves time, especially travel in rural area and allows more regular contact and support.’ Social worker, Mixed acute-community trust

‘You can deal with more people more quickly and [it allows for a] greater monitoring of a wider population.’ Physiotherapist, GP practice

‘Less time-consuming travelling from patient to patient, better for the environment and more time efficient for the patient, as they will not have to travel.’ Paramedic, GP practice

Conversely, some suggested that the cost of the technology would be a barrier to adoption, and that it would be a drain on time to deliver the technology and to manage it:

‘Expensive to provide equipment. Timely and cost to get the equipment to people.’ Occupational therapist, Mental health trust

Many practitioners had the view that digital healthcare could allow for the closer monitoring of patients, which could highlight those at risk of deterioration an earlier point, and allow for the earlier instigation of interventions and the avoidance of hospital admission. This would apply to those who are acutely unwell, as well as those living with longer-term conditions and needing management to prevent frailty:

‘To highlight any changes in a patient’s condition, to ensure they have swifter input and advice when needed to prevent any further deterioration.’ Physiotherapist, Primary care network

‘I think it will be crucial, it will help staff and patients manage the condition better, engage people, and intervene at any earlier stage to prevent/reduce the progression of frailty.’ Physiotherapist, Primary care network

Some suggested that remote monitoring has the potential to provide a truer representation of patients’ observations compared with a single snapshot assessment during a face-to-face visit:

‘Reduced white coat syndrome of variable readings due to staff being present.’ Occupational therapist, Mental health trust

‘Observation monitors as a norm baseline for what is normal for that patient, for example, saturations. Support emergency assessments to identify what is an acute change versus the norm.’ Occupational therapist, Acute trust

Some identified that that this technology could allow for patients/carers/relatives to have greater insight into their health through live feedback, which could empower patients and result in better self-management:

‘There is a great opportunity here to enable people living with frailty to get real-time feedback and support.’ Physiotherapist, Mixed acute-community trust

‘Those without cognitive impairment and literacy in technology, or those with a care giver who is tech-literate, can be empowered to self-manage more, as well as recognise early signs of deterioration.’ Physiotherapist, Acute trust

In contrast to these points, others proposed that technology was perhaps not sensitive enough to highlight subtle symptoms of potential decline, and did not provide clinicians with a true picture of patients’ health compared with face-to-face consultations:

‘Relying on remote assessments may mean some symptoms are overlooked. Often, with frailty, there are comorbidities which need a holistic assessment. You cannot listen to a patient’s chest or feel an abdomen remotely. Patients of an elderly generation are sometimes not forthcoming with symptoms, which are usually found during physical examination. This may be missed if consulting remotely.’ Paramedic, GP practice

‘Not full picture of patients’ condition, missing the “clues”.’ Pharmacist, Primary care provider

### Adopters

The potential benefits of digital healthcare were well documented in the survey responses. However, with some respondents, there were concerns that technology may be a potential replacement to elements of their job, rather than an enhancement:

‘Training and reassurance that digital/remote care is an addition to, not a replacement for, face-to-face assessments/communication.’ Paramedic, Independent sector healthcare provider

For others, introducing technology was yet another change to already stretched services, which have been through considerable upheaval during the past 3 years:

‘Fatigue with further change in practice/protocol following COVID-19. Institutional resistance to changing practice.’ Podiatrist, Mixed acute-community trust

A few believed that some patients and carers may be resistant to change, or do not trust technology, which could impact on engagement and adoption:

‘People refusing technology and/or being deprived of freedom and liberty.’ Nurse, Acute trust

‘Most see technology as a barrier. I feel if left to utilise it independently, they would struggle to engage with it.’ Paramedic, Independent sector healthcare provider

Most respondents suggested that training in using technology, interpreting data and troubleshooting would be required for both staff and patients. Ongoing support may be needed for some patients and their relatives/carers, which may necessitate additional time and staffing:

‘I think there are significant training needs, as the NHS has a very poor record of implementing technology into practice. Most staff will require a significant amount of training to come up to speed with the technology which is available.’ Occupational therapist, Acute trust

‘Advanced practitioners would not have the time to manage/teach the IT/user-end of the technology… this would be a poor use of senior clinician time.’ Nurse, Acute trust

The role of carers/relatives was reported by some as critical in helping certain patients to engage and utilise technology:

‘Very useful. Some relatives already use this sort of digital care to monitor elderly relatives when they are in work—similar to a baby video monitor.’ Nurse, GP practice

‘Carers were usually more technologically minded and able to use zoom; however, patients struggled with this and often missed sessions or were not able to log on.’ Occupational therapist, Mental health trust

### Organisation

Organisational limitations were reported, particularly around information governance, data monitoring and safety. Some reported a lack of confidence in their local IT systems to provide the right type of technology. Some had the view that trusts were perhaps too cautious in adopting technology:

‘Training of staff and the training of trusts regarding risk management/safety [would be necessary,] as many trusts are risk averse when it comes to technology.’ Physiotherapist, Mixed acute-community trust

‘Managing the technology, having technology that is good enough in terms of its specification and durability. I have little confidence that this would happen.’ Physiotherapist, Primary care network

### External context

The prospect of having insufficient geographical coverage for technologies such as 4G, 5G and Wifi was also raised:

‘Many rural areas and patients have no WiFi. Some patients will be unable to use the technology.’ Nurse, Mixed acute-community trust

## Discussion

This is the first UK survey that explored the advanced practice role of those who work with older people and those living with frailty in community and primary care settings in the UK; it is also the first survey to explore the use of technology in such roles. The authors found wide variation in the characteristics of APs and the services they worked in. This is consistent with the findings of a survey of advanced roles across several specialities across several settings (Fothergill et al, 2022); although differences were found in terms of qualifications. While Fothergill et al (2022) reported that 57% of APs were educated to MSc level, the authors found that 65% held this level of qualification, with 81% having at least a postgraduate level qualification. The authors expected that a higher proportion of respondents would be from a nursing background where the AP role has been established for some time, compared with AHPs and other registered health and social work practitioners (Stewart-Lord et al, 2020). Most survey respondents worked at an AP level, with very few working as a consultant or trainee consultant. While medical consultant roles have existed in hospitals since the 1960s (Ministry of Health, 1967), consultant roles are relatively new in the NHS for other professionals, having been introduced for nurses in 1999 (Key, 2000) and for AHPs in 2000 (Department of Health, 2000).

In the qualitative data, the authors identified several multi-faceted complexities associated with introducing technology into health and care for those living with frailty, health and care staff and organisations. These findings may explain why the current use of technology by APs is low. However, a commonly cited barrier was that older people themselves cannot use technology. This was disputed by the authors’ study advisory group of older people, who felt that they were keen and able to use technology, but were either not given the opportunity or were met with a range of reasons as to why it was not possible.

There has been a significant expansion in the availability and use of health technology, particularly in the past few years (Hutchings, 2022). However, this does not apply directly to those aged 75 years and over, 42% of which do not use the internet, and cite a lack of digital skills as the main reason (Age UK, 2021). This said, the population is changing and the use of technology among those aged over 55 years is more common, with 77% reportedly using smart phones (Lee, 2018) and 88% of those aged 50 to 64 years reportedly using the internet most or every day (Age UK, 2021). Reducing digital inequalities among older people will require a range of strategies to give them access to devices and the internet, as well as give them the knowledge and skills to optimise use (Mistry and Jabbal, 2023). This, accompanied by the changing demographics of older people with experience of using technology may increase the acceptability of digital health and social care over the next 10 to 20 years.

The increasing drive from policy to deliver more remote and digitally enabled care (NHS Digital, 2022) and to increase virtual wards for those living with frailty (NHS England and NHS Improvement, 2022) is not without challenges. White this survey found interest from staff in using technology in their practice, there was a lack of availability, training and infrastructure in the implementation of such technologies. To promote digital readiness, the formation of dedicated digital teams have been recommended (Hammerton et al, 2022), along with the implementation of digital skills training (Masoli et al, 2023; Wynn et al, 2023).

## Strengths and limitations

This is the first national survey that examines advanced practice roles of those working with older people outside of hospital settings. Through the use of both closed and free-text questions, the authors were able to gain a deeper understanding of the roles and services the APs worked across, and their views on technology. As the survey was distributed widely using social media and professional networks, the authors were unable to establish a response rate or determine whether the sample was representative. However, participant characteristics were varied, which enabled the authors to capture a broad picture of these roles.

While the completion rate for all closed questions was very high, they were much lower for the open questions. This said, the authors were able to identify common themes from the open responses that were invaluable in informing the development of a prospective community-based model of CGA that could be implementable in UK health and care services.

## Future research

The results of this survey will be used, along with findings from the DREAM study (Mahmoud et al, 2023; Bollen et al, 2024; Whitney et al, 2024), to develop a model of digitally-enhanced CGA for evaluation in a randomised controlled trial.

## Conclusion

Those working in advanced practice roles with older people in community and primary care settings are a wide-ranging group of health and social work professionals in terms of their professional background, qualifications, roles and experiences. While their current use of technology in practice is small, it is likely to be highly valuable in the future, albeit on the basis that its limitations are addressed and implementation can be conducted across health and social care. **IJAP**

## Figures and Tables

**Table 1:** Participant Characteristics

Category	Variable	Frequency (%)
Sex (n=111)	Female	94 (85)
	Male	16 (14)
	Non-binary	1 (1)
Ethnicity (n=110)*	White	103 (93.7)
	Black, Black British, Caribbean or African	3 (2.7)
	Asian or Asian British	2 (1.8)
	Other	2 (1.8)
Professional background (n=110)	Nursing	63 (56.8)
	Physiotherapy	21 (18.9)
	Occupational therapy	15 (13.5)
	Paramedic	5 (4.5)
	Podiatry	4 (3.6)
	Pharmacy	1 (0.9)
	Social work	1 (0.9)
	Other	1 (0.9)
Highest qualification (n=111)	Doctorate	4 (3.6)
	Master’s degree or equivalent	72 (64.9)
	Post-graduate certificate	14 (12.6)
	Undergraduate degree or equivalent	17 (15.3)
	Diploma	4 (3.6)
Years as a registered professional (n=110)	>20	50 (45.5)
	11-15	21 (19.1)
	16-20	19 (17.3)
	6-10	17 (15.5)
	1-5	3 (2.7)

* Ethnic groups based on 2021 Census of England and Wales

**Table 2:** Role Characteristics

Category	Variable	Frequency (%)*
Advanced role (n=110)	Advanced practitioner	58 (52.7)
	Trainee Advanced practitioner	33 (30)
	Consultant practitioner	8 (7.3)
	First contact practitioner	5 (4.6)
	Trainee Consultant practitioner	2 (1.8)
	Other	4 (3.6)
Professional grade (n=111)	NHS Band 8c	2 (1.8)
	NHS Band 8b	13 (11.7)
	NHS Band 8a	38 (34.2)
	NHS Band 7	50 (45.1)
	NHS Band 6	4 (3.6)
	Other	4 (3.6)
Role in team (n=110)	Team lead	16 (14.5)
	Professional lead	27 (24.6)
	Team member	66 (60)
	Multiple roles	1 (0.9)
Employment (n=1111)	Full time	79 (71.1)
	Part time	32 (28.9)
Years in advanced practice role (n=110)	<1	18 (16)
	1 to 5	55 (50)
	6 to 10	16 (14)
	11 to 15	10 (9)
	16 to 20	4 (4)
	>20	1 (1)
	Still in training	6 (6)
Employer (n=111)	Community trust	33 (29.8)
	Acute trust	29 (26.1)
	GP practice or primary care network	23 (20.7)
	Mixed community-acute trust	19 (17.1)
	Mental health trust	3 (2.7)
	Independent health provider	2 (1.8)
	Multiple employers	1 (0.9)
	Local authority	1 (0.9)
Geographical location (n=111)*	Rural and coastal	52 (47)
	Suburban	62 (56)
	Urban	74 (67)
Supervision (n=110) (frequency)	Weekly	11 (10)
	Every 1–4 weeks	35 (32)
	Every 5–10 weeks	36 (33)
	Every few months	6 (5)
	Varies	7 (6)
	Rarely or never	15 (14)

* Respondents could select more than one answer

**Table 3:** Service Characteristics

Category	Variable	Frequency (%)*
Work setting (n=110)*	Patient/client own home	71 (65)
	Care homes	64 (58)
	Hospital	33 (30)
	Outpatient	23 (21)
Service population (n=111)*	Older people	80 (72)
	Older people with frailty	82 (74)
	Adults	39 (35)
	Children and adolescents	1 (0.9)
Services provided (n=111)*	Anticipatory care	49 (41)
	Enhanced care in care homes	39 (35)
	Urgent community response	38 (34)
	Community palliative care	35 (32)
Referral sources (n=111)*	General practitioners	877 (78)
	Community services/teams	82 (74)
	Acute trust teams	77 (69)
	Self-referral	36 (32)
Service leadership (n=82)	Clinical lead (profession unspecified)	25 (30.4)
	Nurse	19 (23.2)
	General practitioner	15 (18.3)
	Geriatrician	15 (18.3)
	Allied Health Professional	5 (6.1)
	Non-clinical manager	3 (3.7)

* Respondents could select more than one answer

**Table 4:** Use Of Technology In Practice

Category	Variable	Frequency (%)
Communication with patients/clients regularly/often using…	Telephone (n=111)	82 (74)
	Text (n=103)	12 (12)
	Videocalls (n=101)	6 (6)
	WhatsApp (n=101)	5 (5)
Remote monitoring regularly/often using…	Blood pressure monitor (n=107)	34 (32)
	Pulse oximeter (n=107)	34 (32)
	Falls sensor (n=107)	9 (8)
	Activity monitor (n=103)	10 (10)
	Digital cognitive assessment (n=105)	10 (10)
	Environmental sensors/monitors (n=104)	8 (8)
Online interventions regularly/often using (n=104)	Live video	1 (1)
	Apps	4 (4)
	Local community platform	3 (3)
	YouTube	0 (0)
	Vimeo	0 (0)

## Data Availability

The datasets used and/or analysed during the current study are available from the corresponding author on reasonable request.

## Key Points

◼ This is the first study examining advanced practitioner (AP) roles in community and primary care settings for older people
◼ These APs are a wide-ranging group of health and social work professionals, with a variety of professional backgrounds, qualifications, roles and experiences
◼ Free-text responses enabled the authors to gain a deeper understanding on the factors that impact on their use of technology
◼ While their current use of technology in practice is small, it is predicted to have a high potential value in the future.

## CPD / Reflective Questions

◼ How do you support older people to engage with technology as part of their health and care?
◼ How could you feel confident using technology in your practice?
◼ How does your organisation support staff and patients to use technology effectively and efficiently in practice?
